# Genomic Analysis of Multidrug-Resistant Hypervirulent (Hypermucoviscous) *Klebsiella pneumoniae* Strain Lacking the Hypermucoviscous Regulators (*rmpA*/*rmpA2*)

**DOI:** 10.3390/antibiotics11050596

**Published:** 2022-04-28

**Authors:** Hisham N. Altayb, Hana S. Elbadawi, Othman Baothman, Imran Kazmi, Faisal A. Alzahrani, Muhammad Shahid Nadeem, Salman Hosawi, Kamel Chaieb

**Affiliations:** 1 Department of Biochemistry, Faculty of Science, King Abdulaziz University, Jeddah 21589, Saudi Arabia; oabaothman@kau.edu.sa (O.B.); ikazmi@kau.edu.sa (I.K.); faisalzh@gmail.com (F.A.A.); mhalim@kau.edu.sa (M.S.N.); shosawi@kau.edu.sa (S.H.); kalshaib@kau.edu.sa (K.C.); 2 Centre for Artificial Intelligence in Precision Medicine, King Abdulaziz University, Jeddah 21589, Saudi Arabia; 3 Microbiology and Parasitology Department, Soba University Hospital, University of Khartoum, Khartoum 11115, Sudan; hanasalah200@gmail.com; 4 King Fahd Medical Research Center, Embryonic Stem Cells Unit, Department of Biochemistry, Faculty of Science, King Abdulaziz University, Jeddah 21589, Saudi Arabia; 5 Laboratory of Analysis, Treatment and Valorization of Pollutants of the Environmental and Products, Faculty of Pharmacy, University of Monastir, Monastir 5000, Tunisia

**Keywords:** antimicrobial resistance, hvKP, K2 capsule, ST14, fimbrial proteins, aerobactin

## Abstract

Hypervirulent *K. pneumoniae* (hvKP) strains possess distinct characteristics such as hypermucoviscosity, unique serotypes, and virulence factors associated with high pathogenicity. To better understand the genomic characteristics and virulence profile of the isolated hvKP strain, genomic data were compared to the genomes of the hypervirulent and typical *K. pneumoniae* strains. The *K. pneumoniae* strain was isolated from a patient with a recurrent urinary tract infection, and then the string test was used for the detection of the hypermucoviscosity phenotype. Whole-genome sequencing was conducted using Illumina, and bioinformatics analysis was performed for the prediction of the isolate resistome, virulome, and phylogenetic analysis. The isolate was identified as hypermucoviscous, type 2 (K2) capsular polysaccharide, ST14, and multidrug-resistant (MDR), showing resistance to ciprofloxacin, ceftazidime, cefotaxime, trimethoprim-sulfamethoxazole, cephalexin, and nitrofurantoin. The isolate possessed four antimicrobial resistance plasmids (*p*KPN3-307_type B, *p*ECW602, *p*MDR, and *p*3K157) that carried antimicrobial resistance genes (ARGs) (*bla*_OXA-1,_ *bla*_CTX-M-15_, *sul2*, *APH(3″)-Ib*, *APH(6)-Id*, and *AAC(6′)-Ib-cr6*). Moreover, two chromosomally mediated ARGs (*fosA6* and *SHV-28)* were identified. Virulome prediction revealed the presence of 19 fimbrial proteins, one aerobactin (*iutA*) and two salmochelin (*iroE* and *iroN*). Four secretion systems (T6SS-I (13), T6SS-II (9), T6SS-III (12), and Sci-I T6SS (1)) were identified. Interestingly, the isolate lacked the known hypermucoviscous regulators (*rmpA/rmpA2*) but showed the presence of other *RcsAB* capsule regulators (*rcsA* and *rcsB*). This study documented the presence of a rare MDR hvKP with hypermucoviscous regulators and lacking the common capsule regulators, which needs more focus to highlight their epidemiological role.

## 1. Introduction

*Klebsiella pneumoniae* is a Gram-negative bacterium associated with invasive hospital-acquired infections [1]. Hypervirulent *K. pneumoniae* (hvKP) overproduces a polysaccharide capsule and is an important clinical pathogen responsible for several infections in healthy and immunosuppressed patients [2,3]. The presence of capsular polysaccharides (CPS) and lipopolysaccharides (LPS) are associated with organism dissemination and virulence [4]. This pathotype with hypermucoviscosity has acquired antimicrobial resistance capable of causing serious invasive disease, unlike the old drug-susceptible strains [3]. The presence of hvKP has been linked to endophthalmitis, pneumonia, liver abscesses, and meningitis [5]. The hvKP phenotype, which contributes to the hypermucoviscous phenotype, is related to the presence of a virulence plasmid containing two capsular polysaccharide regulator genes (*rmpA* and *rmpA2*) as well as multiple siderophore gene clusters and capsular K antigens (*K1*, *K2*, *K5*, *K20*, *K54*, and *K57*) [6,7]. Most of the hvKPs belong to a small collection of clonal groups; the more dominant groups are CG23 and include ST23, 26, 57, and 1633 [8].

Capsules, siderophores, lipopolysaccharides (LPS), fimbriae, outer membrane proteins, and type 6 secretion systems (T6SS) are among the virulence components that contribute to hvKP strains [9]. Most of the hypermucoviscous and hypervirulent strains of *K. pneumoniae* are characterized by the presence of the *rmpA* and *rmpA2* (transcriptional activators, which regulate the mucoid phenotype) regulatory genes [10], but in a few cases, these strains could lack the *rmpA* and *rmpA2* regulators [8,11].

Aerobactin is considered one of the most critical virulence factors in hvKP and is used for the definition of hypermucoviscous strains such as hvKP [6]. Aerobactin-producing isolates are more likely to cause a severe immune response in the host and more invasive infections [6]. In Taiwan, hypermucoviscosity was seen in 88.8% of *K. pneumoniae* isolates from individuals with pyogenic liver abscesses [12]. A purulent liver abscess caused by a very invasive community-acquired *K. pneumoniae* has recently been reported [3]. Furthermore, an outbreak of ST11-type carbapenem-resistant hvKP was reported in a Chinese hospital in 2016 [13].

Most of the hvKPs have remained susceptible to a variety of routinely used antimicrobial agents with the exception of ampicillin, but recently MDR isolates have been increasingly reported worldwide [14,15,16]. Carbapenem-resistant *K. pneumoniae* strains from the clonal group (CG) 258 are the most prevalent, with ST258 and ST11 being the most common multilocus sequence types globally [17]. The acquisition of virulence plasmids by *K. pneumoniae* harboring the insertion of the drug resistance genes *bla*_KPC-2_ and *catA1* has been reported [18,19]. According to Hao et al. [3] the rates of the virulence-associated genes *rmp*A, *iro*B, *fib*, and *hib* were considerably greater in hvKP than in non-hvKP. Furthermore, plasmids carrying two replicons (IncHI1B–IncFIB and IncFIIK–IncFIBK) coding for drug-resistant and virulence genes were discovered [20,21]. The presence of a wide range of β-lactamases, aminoglycoside, and carbapenem-resistant genes could result in the increasing difficulty of treatment and long hospital stays [16,22]. More recently, hvKP belonging to ST147 in COVID-19 patients has been reported in Italy with three plasmid replicons of the IncFIB (Mar), IncR, and IncHI1B types as well as different resistance genes [23]. Additionally, fourteen colistin-resistant *K. pneumoniae* (CoRKp) strains were screened retrospectively in China between 2017 and 2018 [24]. Among them, six CoRKp strains belonging to ST11 were MDR [24]. 

Khartoum is one of the most crowded cities in Africa [25,26] which facilitates the horizontal transfer of antimicrobial-resistant bacteria. Additionally, Sudan suffers from the inappropriate use of antibiotics; most of the antibiotics are frequently sold over the counter and even without a medical prescription [27,28]. In a recent study conducted in Khartoum state, strains positive for β-lactamase and carbapenemase genes have been reported in hvKP isolates [29]. To better understand the genomic characteristics and virulence profile of the newly isolated hvKP strain (named 9KP), this comparative genomic study was conducted. 

## 2. Results

### 2.1. Patient Details and Phenotypic Characterization of the Isolate

The isolate was obtained from a patient with CKD in Soba University Hospital in Sudan, and it was identified with a hypermucoviscous phenotype using the string test, in which mucus is measured more than 9 cm by lop (Supplementary file 1, Figure S1). The isolate was classified according to CLSI breakpoints as MDR when showing resistance to ciprofloxacin, ceftazidime, cefotaxime, trimethoprim-sulfamethoxazole, cephalexin, nitrofurantoin, amoxicillin-clavulanic acid, and ampicillin, while it was susceptible to meropenem, imipenem, amikacin, and gentamicin. A high resistance level was observed for cephalosporins and penicillin, in which a no inhibition zone (0 mm) was observed for amoxicillin-clavulanic acid and ampicillin. Additionally, for the first-generation and third-generation cephalosporins, a small zone of inhibition (10 mm) was observed. Among non-β-lactams, a high resistance level was observed for trimethoprim-sulfamethoxazole (0 mm) and a small zone of inhibition (10 mm) was observed with ciprofloxacin (Table 1). 

For the determination of the minimum inhibitory concentrations (MIC) of the antibiotics, we used the microtitre broth dilution method, which revealed that the isolate possessed a high resistance level against ampicillin (MIC = 1024 μg/mL), tetracycline (MIC = 256 μg/mL), cefotaxime (MIC = 128 μg/mL), and ciprofloxacin (MIC = 128 μg/mL), while two antimicrobial (gentamicin and chloramphenicol) scored a very low MIC (4 μg/mL), falling within the susceptibility range according to CLSI guidelines [30] (Table 1).

### 2.2. Genome Characteristics and Typing

**Figure 1:**
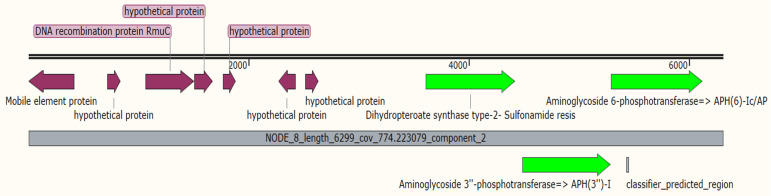
Linear map of *E. coli* plasmid *p*ECW602 which was detected in 9KP strain; the horizontal black lines indicate the length of the plasmid, the middle gray line contains information about plasmid length and coverage. In addition, the purple arrows indicate mobile elements and hypothetical proteins. The green arrows indicate ARGs.

The total genome was assembled into 5364730 bp, with 83 contigs and an average contig length of 64635, while N50 was 220979, L50 7, the average coverage was 100X, and the GC content was 57.3%. The total number of predicted genes was 5248, 76 tRNA, and 202 genes associated with stress response, defense, and virulence (Supplementary file 1, Figure S2). The isolate was identified as *K. pneumoniae* with sequence type (ST) 14 by the Institut Pasteur MLST and MLST 2.0 databases. The global platform for genomic surveillance, Pathogenwatch, was used for the prediction of the capsule (K) and O serotypes; the isolate was identified with the K2 (wzi2 genotype) capsule and O1 serotype. The 9KP strain harbored ten antimicrobial resistance genes including β-lactam resistance genes (*bla*_OXA-1,_ *bla*_CTX-M-15,_ and *bla*_SHV-28_), sulfonamide resistance (*sul2*), fosfomycin resistance (*fosA6*), aminoglycoside resistance (*APH(3″)-Ib*, *APH(6)-Id*, and *AAC(6′)-Ib-cr6*), and the gene causing resistance to tetracycline (*tet(A)*). The chloramphenicol O-acetyltransferase (*CatB3*) gene was detected in the 9KP strain with 70% coverage and 100% identity (Supplementary file 1, Table S1). Additionally, three efflux pumps were identified, including *K. pneumoniae KpnF, LptD,* and *oqxA*. Two chromosomal mutations conferring resistance to fosfomycin (E350Q) and elfamycin EF-Tu (R234F) were also identified. The PlasmidFinder tool revealed the presence of four plasmid replicons (Col440II, IncFII, IncFIB(K), and IncFII(K)) in the 9KP strain with 100% identity and coverage. Additionally, the use of a BLASTn search against the PLSDB database revealed the presence of four plasmids in the 9KP strain, carrying different ARGs, *p*KPN3-307_type B, *p*ECW602, *p*MDR, and *p*3K157, which showed a matching of 99.56%, 99.75%, 100%, and 100%, respectively. The *p*KPN3-307_type B plasmid of the *K. pneumoniae* strain H151440672 was identified in our strain as carrying genes corresponding to *bla*_CTX-M-15,_ RND efflux, and IS1 sequences (Supplementary file 1, Figures S3 and S4). The *Escherichia coli* plasmid *p*ECW602 was detected in the 9KP strain carrying different mobile elements and ARGs-encoding genes, which included sulfonamide (*sul2*) and aminoglycoside resistance genes (*APH(3″)-Ib* and *APH(6)-Id*) (Figure 1). *K. pneumoniae p*MDR was identified with two transposases capturing *tet(A)* MFS-family efflux-pump-encoding genes (Supplementary file 1, Figure S5). Moreover, we detected the chloramphenicol O-acetyltransferase (*CatB3*) gene, class D beta-lactamase (*bla*_OXA-1_), and aminoglycoside N(6′)-acetyltransferase (*aac(6′)-Ib-cr*) genes in the 9KP plasmid (*p*3K157) (Supplementary file 1, Figure S6) while *SHV-28* and fosfomycin resistance (*fosA6*) genes were detected only in chromosomal sequences and were absent in the assembled plasmid, indicating their possible chromosomal association.

One plasmid belonging to the IncFIB(K) type was identified by a BLASTn search against PLSDB and showed 99.7% identity to the *K. pneumoniae* strain SCKP020143 plasmid *p*1_020143, and it was negative for ARGs (Supplementary file 1, Figure S7).

The virulence factor database was used for the prediction and comparison of the virulence genes of the 9KP strain with others. Different types of fimbrial proteins were discovered including type I (10), type 3 (8), and type IV pili (*pilW*) (Table 2) (Supplementary file 1, Table S2). A total of 15 iron uptake proteins were identified, including 1 aerobactin (*iutA*), 12 Ent siderophores, and 2 salmochelin, while it lacked the other aerobactin (*iucA*, *iucB*, *iucC*, and *iucD*) reported in the hvKP strains (NTUH-K2044 and KCTC 2242). The most closely related strains (kkp066 and kkp0e7) were positive for the hvKP marker, the *RmpA* gene, and lacked aerobactin (*iucA*, *iucB*, *iucC*, and *iucD*), similar to our strain. High similarity in the iron uptake system of 9KP and the other Sudanese strain (23KE) was observed, including the complete absence of genes related to yersiniabactin and the presence of two salmochelin and one aerobactin. Four secretion systems that are crucial virulence factors of pathogenic bacteria were identified in the 9KP strain, including T6SS-I (13), T6SS-II (9), T6SS-III (12), and one Sci-I T6SS exclusively detected in our strain. The isolate was positive for two *RcsAB* (*rcsA* and *rcsB*) regulatory proteins and one serum resistance LPS protein. The mediator of the hyper adherence *YidE* in enterobacteria and its conserved region were predicted in the isolate.

### 2.3. Comparative Genomics and Phylogenomics Analysis 

After the genome comparison, the species formed 6142 protein clusters, 3185 orthologous, and 2957 single-copy gene clusters. 9KP showed 192 single-copy genes and 4843 proteins clustered with others (Supplementary file 1, Table S3). A high degree of variability was observed at different chromosomal regions of 9KP, which contains ARGs, incF plasmid proteins, IS, and other mobile elements.

**Figure 2:**
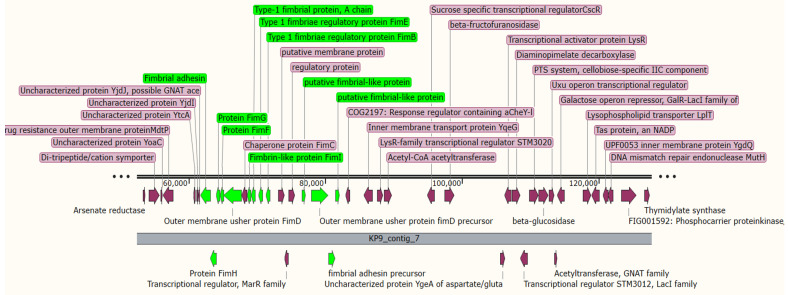
Clustering of fimbrial proteins in contig 7 of *K. pneumoniae* 9KP; the horizontal black lines indicate the contig length, the green arrows indicate genes encoding fimbrial proteins, and the purple arrows indicate other genes located at the same contig.

**Figure 3:**
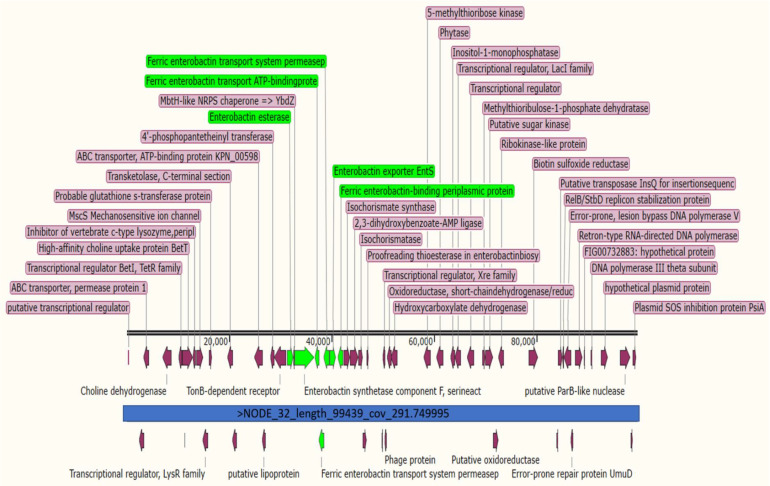
Clustering enterobactin proteins in contig 32 of *K. pneumoniae* 9KP; the horizontal black lines indicate the contig length, the green arrows indicate genes encoding enterobactin proteins, and the purple arrows indicate other genes located at the same contig.

Comparative genomics revealed that the strains TCC BAA-2146, 23KE, kkp066, kkp0e6, and NTUH-K2044 exhibited a high similarity to 9KP, in which different virulent regions were similar, such as the outer membrane protein OmpN, LysR-*type* transcriptional regulators, kinase, and fimbrial proteins (Figure 2) detected at a region located between the chromosomal range 1.5–1.6 Mb. Ferric enterobactin-related proteins and phage-related proteins were clustered in *K. pneumoniae* 9KP similarly to the strains ATCC BAA-2146 and NTUH-K2044 (Figure 3), while the secretion systems T6SS were located in a region adjacent to the VgrG protein, transposases, putative kinase, mobile elements, transcriptional regulator, LysR family, and phage proteins. The PTS system in the 9KP strain was most similar to the PTS system of the 23KE strain from Sudan others (Supplementary file 1, Figure S3, and Supplementary file 2).

**Figure 4:**
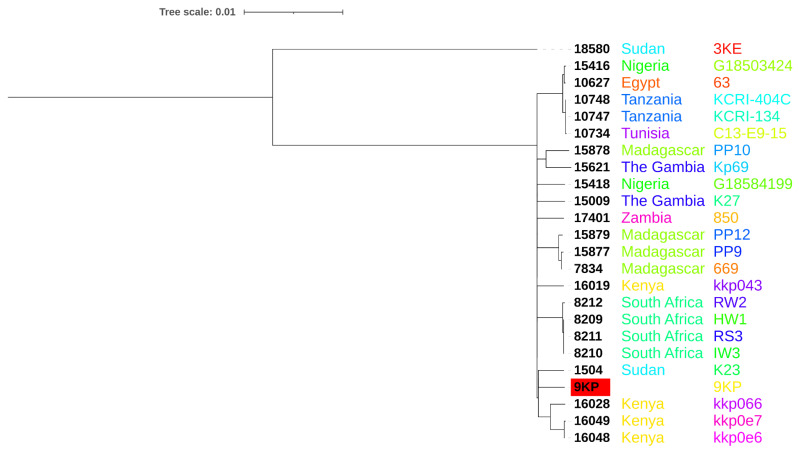
Phylogenomics analysis of *K. pneumoniae* 9KP (shown in red highlight) compared to African *K. pneumoniae* strains. Strain 3KE is *K. quasipneumoniae* used as an outgroup. Numbers in nodes indicate Pasteur MLST isolate IDs.

A phylogenetic tree was generated among the African strains by the iTOL—Interactive Tree of Life—*Klebsiella* Pasteur MLST database. The 9KP strain was clustered in a clade containing three strains from Kenya, one was isolated from a patient with a soft tissue infection (kkp066) and the others (kkp0e6 and kkp0e7) were isolated from hospital environment. And it was also clustered to one MDR Sudanese strain (K23) isolated from drinking water in Khartoum state (Figure 4). 

## 3. Discussion

Hypervirulent *K. pneumoniae* strains possess distinct morphological and genotypic characteristics when compared to other classical strains, which include the production of colonies with hypermucoviscosity, unique serotypes, and virulence factors associated with high pathogenicity [31]. Except for ampicillin, most of the hvKPs have remained susceptible to a variety of routinely used antimicrobial drugs, but recently MDR isolates have been increasingly reported worldwide [14,15,16]. The present study reported MDR hvKP in a patient with a recurrent UTI, and it harbored genes conferring resistance to β-lactam (*bla*_OXA-1,_ *bla*_CTX-M-15,_ and *bla*_SHV-28_), sulfonamide (*sul2*), fosfomycin (*fosA6*), and aminoglycoside (*APH(3″)-Ib, APH(6)-Id*, and *AAC(6′)-Ib-cr6*). The presence of a wide range of β-lactamases and aminoglycoside-resistant genes could result in the increased difficulty of treatment and long hospital stays [16,22]. *Klebsiella* species are known to have intrinsic resistance to ampicillin [32], and here we reported a very high resistance level to ampicillin (MIC ≥ 1024 μg/mL). This could be a result of the presence of additional beta-lactamases (*bla*_CTX-M-15,_ *bla*_OXA-1,_ and *bla*_SHV-28_). A high resistance level was also observed against cefotaxime (MIC ≥ 128 μg/mL), which could be attributed to the presence of *bla*_CTX-M-15_ which possesses a high hydrolytic activity against cefotaxime [33]. Although the isolate harbored chloramphenicol O-acetyltransferase (*CatB3*), the isolate was highly susceptible to chloramphenicol. This could be due to the truncation of the gene, which only showed 70% coverage to the references. 

Our isolate harbored an IncF plasmid, insertion sequences, and phage-associated proteins at regions containing ARGs and virulence genes, which reflect their possible role in the horizontal gene transfer and dissemination of such strains [16]. The IncF plasmids are thought to play a significant role in the acquisition of MDR genes [34,35], which could increase the chance for the acquisition of genes such as the *bla*_KPC_ carbapenem resistance gene.

We identified four plasmids that carried different ARGs and transposases. The presence of the ARGs plasmids in the hvKP strain, which is known to be a more drug-susceptible strain [36], could be a reason for the presence of the MDR phenomenon in our isolate. Additionally, these plasmids may result in the mobility of these ARGs to drug-susceptible isolates. 

Our isolate harbored a *p*KPN3-307_type B plasmid that carried genes corresponding to *bla*_CTX-M-15,_ RND efflux, and IS1 sequences; similar plasmids carrying *bla*_CTX-M-15_ with transposases have been reported in the KPC-producing *K. pneumoniae* ST307 strain in the UK [37]. The presence of the *CTX-M* gene in the mobile elements could be the reason for the current dissemination of the *CTX-M*-positive isolates in our region [38,39]. Moreover, the isolate possessed the heavy metal (copper(I)/silver(I)) efflux pump (RND efflux); isolates resistant to silver have more affinity to establishing hospital and environmental outbreaks [40]. Interestingly, the 9KP strain harbored the plasmid *p*ECW602, which is a novel plasmid reported recently in an extensively drug-resistant (XDR) *E. coli* isolate in China [41]; here we reported it for the first time in a *K. pneumoniae* (9KP) isolate with high identity (99.75%) and high coverage (744). The 9KP plasmid (*p*ECW602) and *E. coli p*ECW602 plasmid carried a similar pattern regarding the presence of sulfonamide (*sul2*) and aminoglycoside resistance genes (*APH(3″)-Ib* and *APH(6)-Id*). The gene responsible for the resistance to tetracycline (*tetA*) associated with the MFS family efflux pump was identified in the *K. pneumoniae* 9KP strain *p*MDR plasmid; the gene expression of the MFS-type *tetA* has been documented in different Gram-negative isolates [42,43]. tet(A)-bearing K. pneumoniae was reported with a high tetracycline and tigecycline resistance level [42]. Adding to that, another tetracycline resistance efflux (*oqxA*) was discovered in our isolate [44]. In addition to plasmid-mediated ARGs, two genes (*fosA6* and *SHV-28)* were not detected among the assembled plasmids of the 9KP strain but they were present in the chromosomes; the fosfomycin resistance gene (*fosA6)* and the broad spectrum B-lactamase *SHV-28* gene are commonly reported in *K. pneumoniae* chromosomes [45,46,47,48]. 

The isolate lacked the common regulators of the hypermucoviscous phenotype *(rmpA*/*rmpA2*) [49] and yersiniabactin system but showed the presence of aerobactin-(*iutA*) and salmochelin-(*iroE* and *iroN*) encoding genes, which are clear markers for hvKP identification [50]. Additionally, the strain was predicted with the K2 capsule type and hypermucoviscosity, which are common virulence factors in hvKP [51]. Similarly, strains belonging to hvKP and lacking the *rmpA* and *rmpA2* genes were previously reported without knowledge of the mechanisms of capsule overexpression [52,53]. One possible explanation of the mucoviscosity in *K. pneumoniae* 9KP is the presence of the *RcsA* and *RcsB* genes; the *RcsA* gene binds with *RcsB* to activate the genes responsible for capsular polysaccharide production in *E. coli* [54]. Another explanation for the presence of the siderophore receptors without biosynthetic genes in hvKP is that these strains can acquire the siderophores from other bacteria found in the same environment [8]. Similar to our finding, a highly virulent and invasive *K. pneumoniae* strain possessing genes such as aerobactin (*iutA*), hypermucoviscosity, salmochelin, and lacking *rmpA*/*rmpA2* was reported in a patient suffering from necrotizing soft tissue infection at Northwestern Memorial Hospital, USA [51]. 

In this study, four T6SS systems were detected. The type VI secretion system (T6SS) is usually located at the chromosomes or pathogenicity islands of virulent bacteria, and they have a role in host infection and colonization [55]. Additionally, eight type 3 fimbrial proteins were reported. Usually, isolates that express type 3 fimbriae are more biofilm-producing compared to other strains [56]. Biofilm-producing isolates can cause community or hospital infections and are associated with 65% of microbial infections and 80% of chronic infections globally [57]. Furthermore, the genomic analysis of the *K. pneumoniae* 9KP strain demonstrated a large abundance of LysR-family transcriptional regulators in the genomic regions containing a cluster of virulence and antimicrobial resistance genes. *LysR* is found in different bacterial species and has a role in the regulation of virulence factors in pathogenic bacteria [58]. A novel type of the LysR family has been demonstrated to have a pleiotropic role in mediating the resistance and increasing the virulence of the hvKP NTUH-K2044 strain [59]. 

The phylogenetic analysis showed that the 9KP strain is more related to strains from Kenya and Sudan. This could be due to the fact that Kenya is a neighboring country to Sudan, and the Sudanese clustered isolate was from the same location (Khartoum) of the sample collection in our study. Two of the Kenyan strains (kkp066 and kkp0e7) were hvKPs possessing the *RmpA* gene and lacked aerobactins (*iucA*, *iucB*, *iucC* and *iucD*), similar to our strain. Additionally, the 9KP strain showed a high similarity in the PTS system to the 23KE strain from Sudan. This could be one of the reasons behind their high similarity to our strain.

MDR and hvKP strains previously developed in distinct clonal groups [60] but the recent emergence of hvKP isolates carrying MDR genes needs more attention. Such a strain has the potential to produce fatal hospital outbreaks, so more focus is needed to highlight its epidemiological role.

## 4. Methods

### 4.1. Bacterial Isolation, Identification, Susceptibility Testing, and DNA Extraction

*Klebsiella* spp. was isolated from the urine sample of a 40-year-old male patient with a history of recurrent UTI, hypertension, and chronic kidney disease (CKD) admitted for hemodialysis in Soba Hospital, Khartoum in July 2021. The patient was visiting the dialysis unit regularly 2 times in a week; the patient received a course of ciprofloxacin twice daily for 3 days without a response. The bacterium was isolated using a MacConkey and blood agar (HiMedia, Mumbai, India), then was identified using routine conventional microbiology methods [61] and Chromogenic UTI media (bioMérieux, Lyon, France). The isolate was identified as a hypermucoviscous strain using the string test [62]. Antimicrobial susceptibility testing was performed using the disk diffusion method to test the activity of amoxicillin-clavulanate (30 µg), cefuroxime (30 µg), ceftriaxone (30 µg), ceftazidime (30 µg), cephalexin (30 µg), meropenem (10 µg), imipenem (10 µg), amikacin (30 µg), gentamicin (10 µg), ciprofloxacin (5 µg), trimethoprim-sulfamethoxazole (25 µg), and nitrofurantoin (300 μg). *K. pneumoniae* ATCC 700603 was used for testing the quality of the culture media, antibiotic disc, and MIC. CLSI guidelines [30] were used for the susceptibility test results interpretation. DNA was extracted using the quinidine chloride protocol [63]. The gel electrophoresis and Nanodrop, Qubit (Thermo Scientific TM, Carlsbad, CA, USA), were used for the estimation of the integrity and quantification of the extracted DNA.

### 4.2. Minimum Inhibitory Concentration (MIC)

The microtitre broth dilution method [64] was used to determine the minimum inhibitory concentration of ciprofloxacin, gentamicin, cefotaxime, ampicillin, chloramphenicol, and tetracycline. A two-fold serial dilution of the antibiotics was prepared in Muller–Hinton (MH) broth, and 100 μL of overnight-grown bacteria adjusted to 5–10^5^ CFU/mL was poured into each well. The antibiotics concentration used was in the range of 2 to 1024 μg/mL [65]. MIC results were interpreted according to CLSI guidelines [30]. 

### 4.3. Genome Sequencing and Assembly

Whole-genome sequencing was conducted by Novogene Company (Beijing, China) using HiSeq 2500 platform (Illumina, San Diego, CA, USA). The generated short reads (2 × 150 bp) were assembled into contigs using a de novo assembly of Velvet v. 1.2.10 [66]; then, reads with low quality and less than 200 bp were removed. The assembled sequences were submitted to GenBank under bioproject (PRJNA767482), biosample (SAMN26332310), and accession number JAKWFM000000000, and were assigned the 9KP strain. The isolate was identified using MLST 2.0 and the Pasteur MLST. The PATRIC web server and the NCBI Prokaryotic Genome Annotation Pipeline (PGAP) [67] were used for genome annotation. 

### 4.4. Plasmid Assembly and Identification

The plasmidSPAdes tool v3.15.4 [68] was used for the assembly of the putative plasmids sequences from the illumine short read, using different k-mer sizes (21, 33, and 55). The generated plasmids were further evaluated by the Plasmid Finder 2.1 tool using 95% identity and 60% coverage. Additionally, the generated plasmids were aligned using BLASTn against the plasmid sequences obtained from the plasmid database (PLSDB); then, a local database of the obtained plasmids was generated at OmicsBox v2.1, and a local blast search was used for the identification of the plasmids. A plasmid circular map was generated by the SnapGene Viewer 6.0.2 software.

### 4.5. Identification of Antimicrobial-Resistant Genes (ARGs) and Mobile Elements

To identify plasmid-mediated ARGs, the generated plasmids were submitted to the Resistance Gene Identifier (RGI) 5.2.1 and ResFinder 4.0 [69] databases; hits with ≥95% identity and ≥98% coverage were accepted. Furthermore, ResFinder 4.0 was used to detect chromosomal mutations conferring resistance to antibiotics; this tool contains a hit that can be flagged to indicate whether the hit is a plasmid or chromosomally mediated. Insertion sequences (IS) were identified by an IS Finder. 

### 4.6. Prediction and Comparison of Virulence Genes

The virulence factors of the hvKP strain were screened using RAST 2.0 and the virulence factor database (VFDB) [70]. The capsule-type genes were identified using the Kleborate v2.2.0 [71] and Pathogenwatch database. The isolate (9KP) virulence profile was compared to a list of *K. pneumoniae* strains including the most closely related strains (23KE, kkp066, kkp0e6, and kkp0e7) and those found in the VFDB database which includes *K. pneumoniae* 342, MGH78578, NTUH-K2044, 1084, HS11286, KCTC 2242, and SB3432; among these strains, two (NTUH-K2044 and KCTC 2242) were hvKP [72]. SnapGene Viewer v.6.0.2 (GSL Biotech; available at snapgene.com, accessed on 20 March 2022) was used for the visualization of the virulence genes cassettes.

### 4.7. Comparative Genomics and Phylogenetic Analysis

The PATRIC v3.6.12 proteome comparison tool [73] was used to perform a protein-sequence-based genome comparison using bidirectional BLASTp. The OrthoVenn2 server [74] was used for protein orthologous clustering analysis. The most closely related genomes (23KE, kkp066, and kkp0e7) and the commonly used strains (*K. pneumoniae* BAA2146, HS11286, MGH78578, NTUH-K2044, NUHL24835, and PittNDM01) for *K. pneumoniae* genome comparison [31,75,76] were used as references. The phylogenetic tree was generated and visualized by the online Interactive Tree of Life (iTOL v6) tool available at Pasteur MLST. This tool generates neighbor-joining trees from concatenated nucleotide sequences; we considered all loci that contained allele sequence identifiers and cgMLST schemes for tree generation. The tree was generated against the most similar African strains of *K. pneumoniae* submitted to the Pasteur MLST database.

## 5. Conclusions

This study documented the presence of a rare MDR hvKP, *K. pneumoniae* 9KP, belonging to *K2* and ST14 with hypermucoviscous; it lacked the yersiniabactin system and the common regulators (*rmpA*/*rmpA2*) of the hypermucoviscous but showed the presence of other capsule regulators, such as *RcsAB* (*rcsA* and *rcsB*) and aerobactin (*iutA*), as well as the presence of salmochelin-(*iroE, iroN*) encoding genes, which are clear markers for hvKP identification.

The MIC revealed that the isolate possessed a high resistance level against ampicillin (1024 μg/mL), tetracycline (256 μg/mL), cefotaxime (128 μg/mL), and ciprofloxacin (128 μg/mL).

The isolate possessed four antimicrobial resistance plasmids (*p*KPN3-307_type B, *p*ECW602, *p*MDR, and *p*3K157) that carried different ARGs and transposases, indicating their possible horizontal transfer and the clonal spread. The *p*ECW602 plasmid is a novel plasmid reported recently in an extensively drug-resistant (XDR) *E. coli* isolate in China [41]; here, for the first time, we reported it in a *K. pneumoniae* (9KP) isolate with high identity (99.75%).

## Figures and Tables

**Table 1:** Antimicrobial susceptibility testing of selected antimicrobial agents used against 9KP strain.

Antibiotic	Inhibition Zone (mm)	MIC (μg/mL)	Susceptibility ^a^
ciprofloxacin	12	128	R
ceftazidime	10	-	R
cefotaxime	10	128	R
trimethoprim-sulfamethoxazole	No inhibition	-	R
cephalexin	10	-	R
nitrofurantoin	10	-	R
amoxicillin-clavulanic acid	No inhibition	-	R
ampicillin	No inhibition	1024	R
tetracycline	-	256	R
meropenem	32	-	S
imipenem	30	-	S
amikacin	20	-	S
gentamicin	20	4	S
chloramphenicol	-	4	S

Abbreviation: R = Resistant, S = Sensitive, - = Not tested, mm = millimeter; ^a^ Antimicrobial susceptibility testing determined according to CLSI guidelines [30].

**Table 2:** Comparison of virulence factors of *K. pneumoniae* 9KP with other control strains (*K. pneumoniae* 342, MGH 78578, NTUH-K2044, 1084, HS11286, JM45, KCTC 2242, SB3432) and the most related strains (kkp066, kkp0e6, and 23KE).

Virulence Factor	Related Genes	9KP	342	MGH78578	NTUH-K2044	1084	HS11286	JM45	KCTC 2242	SB3432	kkp066	kkp0e6	kkp0e7	23KE
Adherence														
Type 3 fimbriae	8	+	+	7	+	+	+	+	+	+	+	+	7	+
Type I fimbriae	10	+	+	+	+	+	+	+	+	+	9	9	8	+
Type IV pili	12	1	-	-	-	-	-	-	-	-	-	-	-	-
Antiphagocytosis														
Capsule	1	+	+	+	+	+	+	+	+	+	+	+	+	+
Efflux pump														
AcrAB	2	+	+	+	+	+	+	+	+	+	1	+	+	+
Iron uptake														
Aerobactin	5	1	1	1	+	1	1	1	+	+	1	1	1	1
Ent siderophore	13	12	+	+	+	+	+	12	+	-	12	10	11	+
Salmochelin	5	2	2	2	+	4	2	2	2	4	2	2	2	2
Yersiniabactin	11	-	-	-	+	+	+	-	-	-	-	+	+	-
Nutritional factor														
Allantoin utilization	6	-	-	-	+	+	-	-	-	-	+	-	-	-
Regulation														
RcsAB	2	+	+	+	+	+	+	+	+	+	+	+	+	+
RmpA	1	-	-	-	+	-	-	-	+	-	1	-	1	-
Secretion system														
T6SS-I	18	13	11	11	13	13	+	+	+	10	16	15	15	12
T6SS-II	10	9	+	8	1	1	1	1	-	4	1	1	-	1
T6SS-III	18	12	+	11	14	13	14	13	14	11	10	8	5	12
Sci-I T6SS	27	1	-	-	-	-	-	-	-	-	-	-	-	-
Serum resistance														
LPS rfb locus	1	+	+	+	+	+	+	+	+	+	+	+	+	-
Toxin														
Colibactin	18	-	-	-	-	+	-	-	-	-	-	-	-	+

Key: + means the presence of the same number of genes, - means gene absent, numbers in tables indicate numbers of virulence-factors-related genes.

## Data Availability

The data for this project was submitted to GenBank under the Bioproject PRJNA767482 and in the additional files.

## Supplementary Materials

The following are available online at https://www.mdpi.com/article/10.3390/antibiotics11050596/s1, Supplementary file 1: contains Tables S1–S3, representing ARGs (Table S1), virulence factors (Table S2), and numbers of the clustered and singletons proteins in the 9KP strain compared to others (Table S3). Additionally, it contains figures from Figures S1–S7 representing the string test photograph (Figure S1), a pie chart of the annotated subsystem and genes of *K. pneumoniae* 9KP (Figure S2), a circular map of the whole-genome comparison of the 9KP strain to different *K. pneumoniae* strains (Figure S3), and a map of the *K. pneumoniae* strain 9PK plasmids (Figures S4–S7),). Supplementary file 2: contains the complete data of the whole-genome comparison of the 9KP strain to different *K. pneumoniae* strains.
