# Supplementary material for: Genomic Analysis of Multidrug-Resistant Hypervirulent (Hypermucoviscous) Klebsiella pneumoniae Strain Lacking the Hypermucoviscous Regulators (rmpA/rmpA2)
Source: Antibiotics (Basel). 2022 Apr 28;11(5):596. doi: 10.3390/antibiotics11050596 (PMC9137517; doi:10.3390/antibiotics11050596)
Supplement: Supplementary file 1 [file antibiotics-11-00596-s001.zip › Supplementary Files 1.pdf]

Table S1. Predicted antimicrobial resistance genes in *K. pneumoniae* 9KP WGS data

Gene name	Accession	contig	Position in genomic contig	Coverage	Identity
sul2	AY034138	48	3467-4282	100%	100%
aph(6)-Id	M28829		5146-5982	100%	100%
aph(3'')-Ib	AF321551		4343-5146	100%	100%
bla_{SHV-28}	AF299299	1	27860-28720	100%	99.88%
fosA6	ACWO01000079	4	333936-334355	100%	99.28%
aac(6')-Ib-cr	DQ303918		1541-2140	100%	100%
bla_{OXA-1}	HQ170510		1410-580	100%	100%
tet(A)	AJ517790	9	2135-3334	100%	100%
*CatB*3	AJ009818		11563..12738	70%	100%

Table S2. Predicted virulence factors in *K. pneumoniae* 9KP

Adherence	Related Gene	Accession Number	Antiphagocytosis	Accession Number	Iron uptake	Related Gene	Accession Number	Secretion system	Related Gene	Accession Number

Type 3 fimbriae	MrkA	MCH6120634.1		MCH6118948.1	Aerobactin	IutA	MCH6121095.1	T6SS-I	clpV/tssH	MCH6119609.1
Type 3 fimbriae	MrkB	MCH6120635.1		MCH6118949.1	Ent siderophore	EntA	MCH6122808.1	T6SS-I	dotU/tssL	MCH6119606.1
Type 3 fimbriae	MrkC	MCH6120636.1		MCH6118950.1	Ent siderophore	EntB	MCH6122807.1	T6SS-I	hcp/tssD	MCH6119608.1
Type 3 fimbriae	MrkD	MCH6120637.1		MCH6118951.1	Ent siderophore	EntC	MCH6122805.1	T6SS-I	icmF/tssM	MCH6119618.1
Type 3 fimbriae	MrkF	MCH6120638.1	Capsule	MCH6118952.1	Ent siderophore	EntD	MCH6122996.1	T6SS-I	impA/tssA	MCH6119619.1
Type 3 fimbriae	MrkH	MCH6120641.1		MCH6118955.1	Ent siderophore	EntE	MCH6122806.1	T6SS-I	ompA	MCH6119607.1
Type 3 fimbriae	MrkI	MCH6120640.1		MCH6118956.1	Ent siderophore	EntS	MCH6122803.1	T6SS-I	sciN/tssJ	MCH6119623.1
Type 3 fimbriae	MrkJ	MCH6120639.1		MCH6118957.1	Ent siderophore	FepA	MCH6122328.1	T6SS-I	tssF	MCH6119621.1
Type I fimbriae	FimA	MCH6120627.1		MCH6118960.1	Ent siderophore	FepB	MCH6122804.1	T6SS-I	tssG	MCH6119622.1

Type I fimbriae	FimB	MCH6120629.1		MCH6118961.1	Ent siderophore	FepC	MCH6122800.1	T6SS-I	vasE/tssK	MCH6119605.1
Type I fimbriae	FimC	MCH6120625.1		MCH6118962.1	Ent siderophore	FepD	MCH6122802.1	T6SS-I	vgrG/tssI	MCH6119610.1
Type I fimbriae	FimD	MCH6120309.1		MCH6118963.1	Ent siderophore	FepG	MCH6122801.1	T6SS-I	vipA/tssB	MCH6119603.1
		MCH6120624.1		MCH6118965.1	Ent siderophore	Fes	MCH6122994.1	T6SS-I	vipB/tssC	MCH6119604.1
Type I fimbriae	FimE	MCH6120628.1		MCH6122094.1	Salmochelin	IroE	MCH6118329.1	T6SS-II	clpV	MCH6122656.1
Type I fimbriae	FimF	MCH6120623.1			Salmochelin	IroN	MCH6119501.1	T6SS-II	dotU	MCH6120746.1
Type I fimbriae	FimG	MCH6120622.1	**Serum resistance**	**Accession**	**Regulation**	**Gene**	**Accession**	T6SS-II	icmF	MCH6120739.1
Type I fimbriae	FimH	MCH6120621.1	LPS rfb locus	MCH6118939.1	RcsAB	RcsA	MCH6120814.1	T6SS-II	impF	MCH6120735.1
Type I fimbriae	FimI	MCH6120626.1		MCH6118940.1	RcsAB	RcsB	MCH6119087.1	T6SS-II	impH	MCH6120737.1
Type I fimbriae	FimK	MCH6120620.1		MCH6118941.1	**Efflux pump**	**Gene**	**Accession**	T6SS-II	impJ	MCH6120747.1

Type IV pili	PilW	MCH6123001.1

MCH6118942.1	AcrAB	AcrA	MCH61211998.1	T6SS-II	ompA	MCH6120745.1
MCH6118943.1	AcrAB	AcrB	MCH61211383.1	T6SS-II	sciN	MCH6120736.1
MCH6118944.1				T6SS-II	vgrG	MCH6120744.1
MCH6118945.1						

Table S3. Overview of the clustered and singletons proteins among different species used in comparison with *K. pneumoniae* 9KP

**Species**	**Proteins**	**Clusters**	**Singletons**
K. pneumoniae BAA_2146	4600	4410	154
K. pneumoniae 9KP	5064	4843	192
K. pneumoniae HS11286	5779	5185	495
K. pneumoniae MGH_78578	8134	4754	2016
K. pneumoniae NTUH_K2044	6232	5113	458
K. pneumoniae NUHL24835	5292	5087	166
K. pneumoniae PittNDM01	5422	4976	346

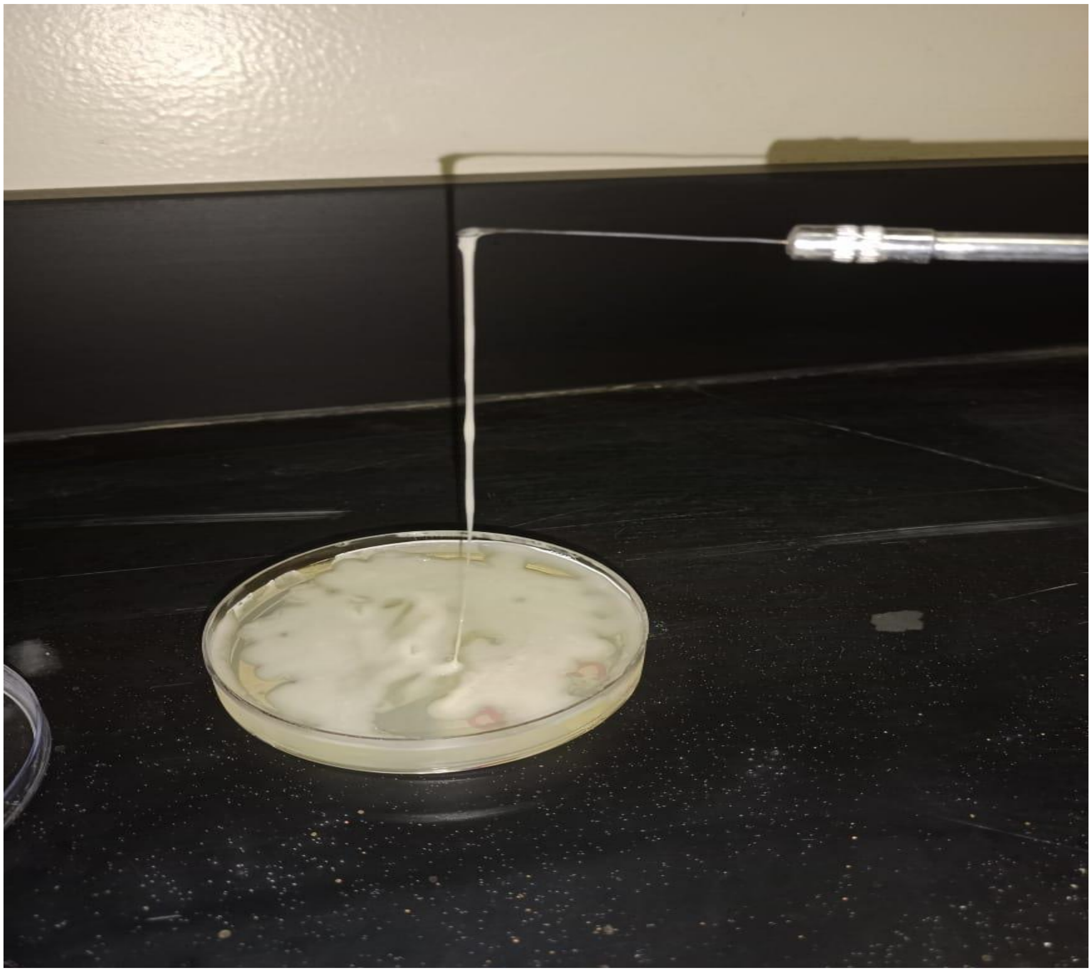

Figure S1. String test showing hypermucoviscocity of 9KP strain.

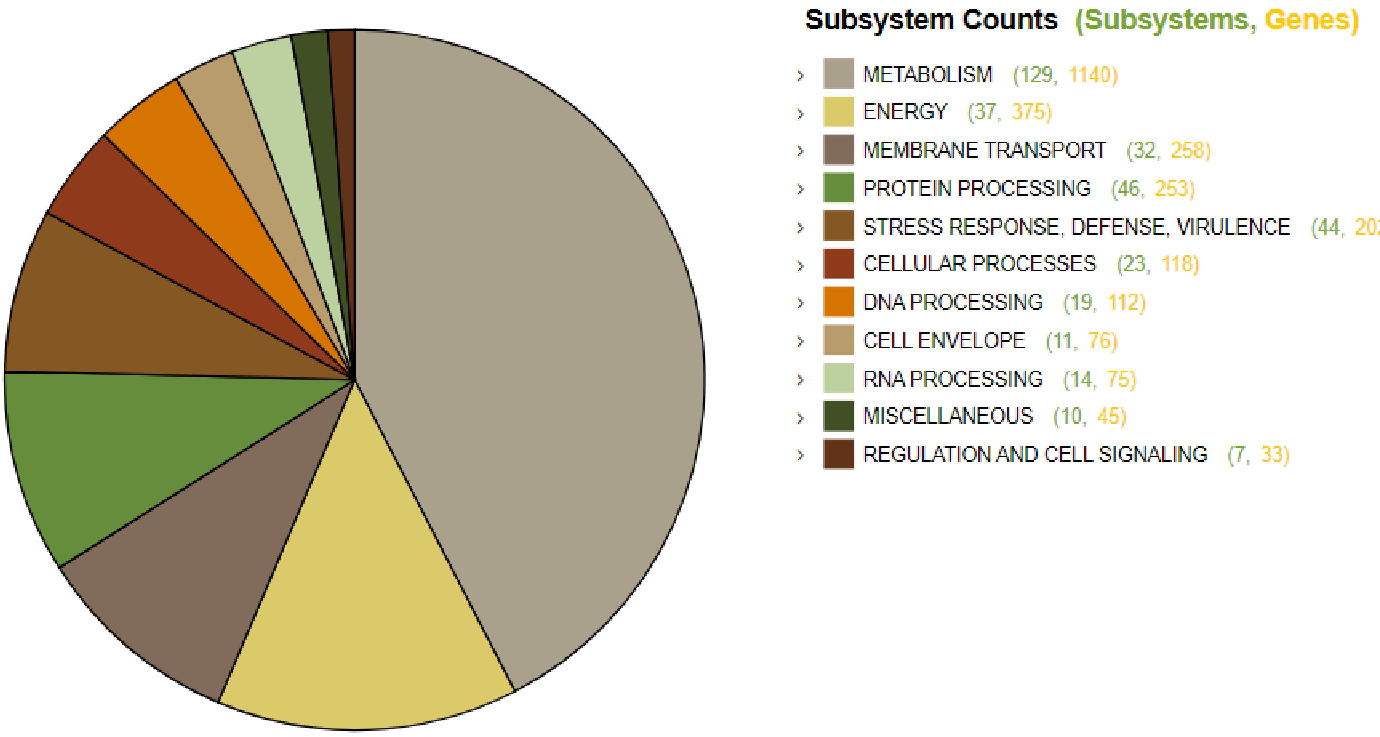

Figure S2. Pie chart of the annotated subsystem and genes of *K. pneumoniae* 9KP

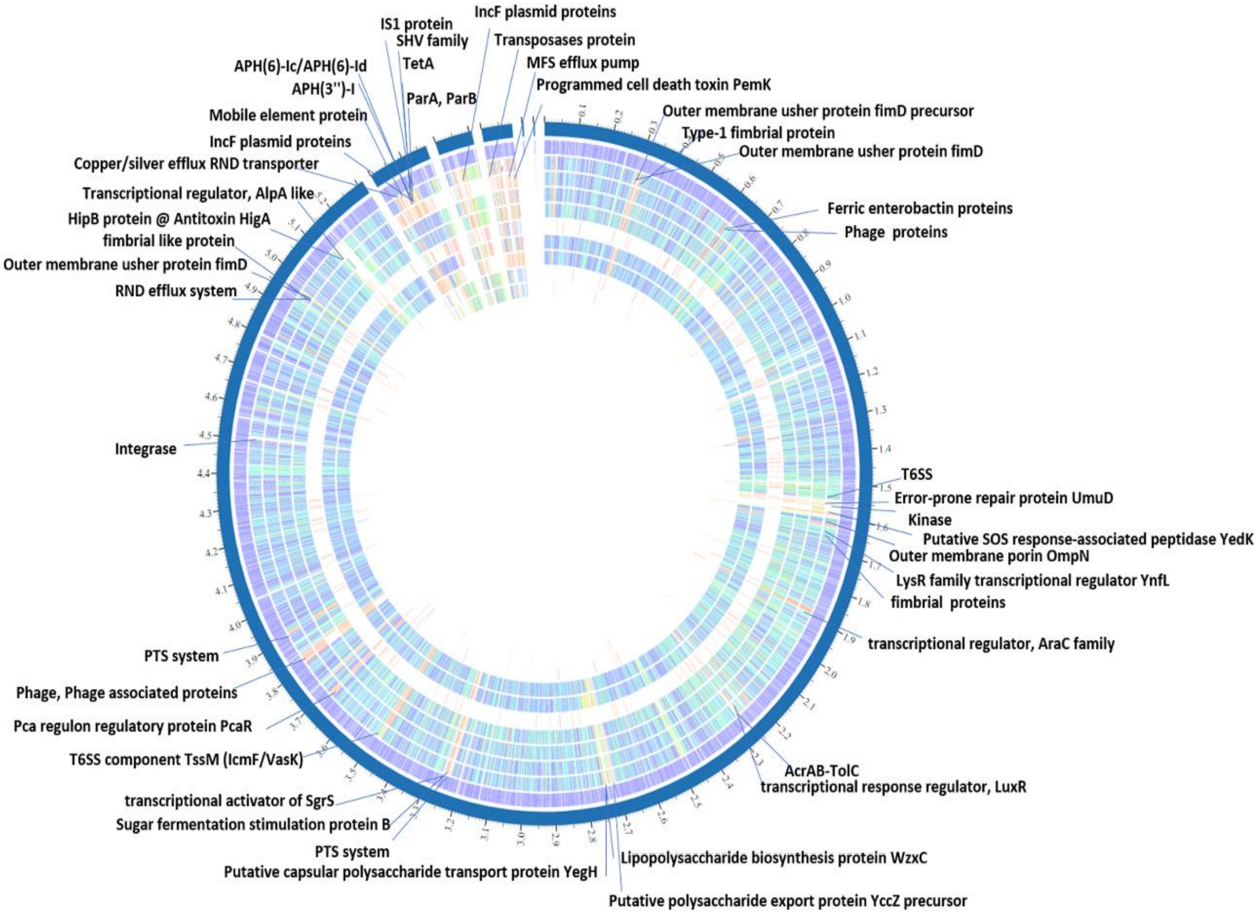

Figure S2. Whole-genome comparison of different *K. pneumoniae* control strains with *K. pneumoniae* 9KP, showing regions with a low protein sequence identity of 9KP (in red and yellow colors), while blue colors indicate high similarity. List of tracks, from outside to inside: MGH 78578, 9KP, 23KE, kkp066, kkp0e7, PittNDM01, ATCC BAA-2146, NTUH-K2044, HS11286, and NUHL24835.

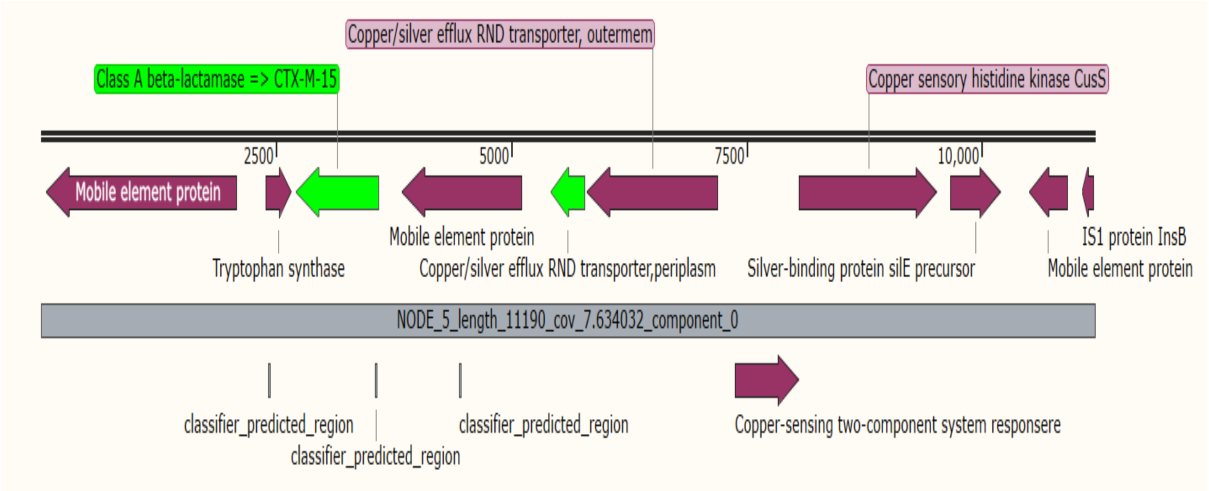

Figure S4. Map of *K. pneumoniae* plasmid *p*KPN3-307 type B; the horizontal line indicates the length of the plasmid, the middle gray line contains information about plasmid length, and coverage, and the purple arrows indicate mobile elements, the green arrows indicate ARGs, and efflux pump genes.

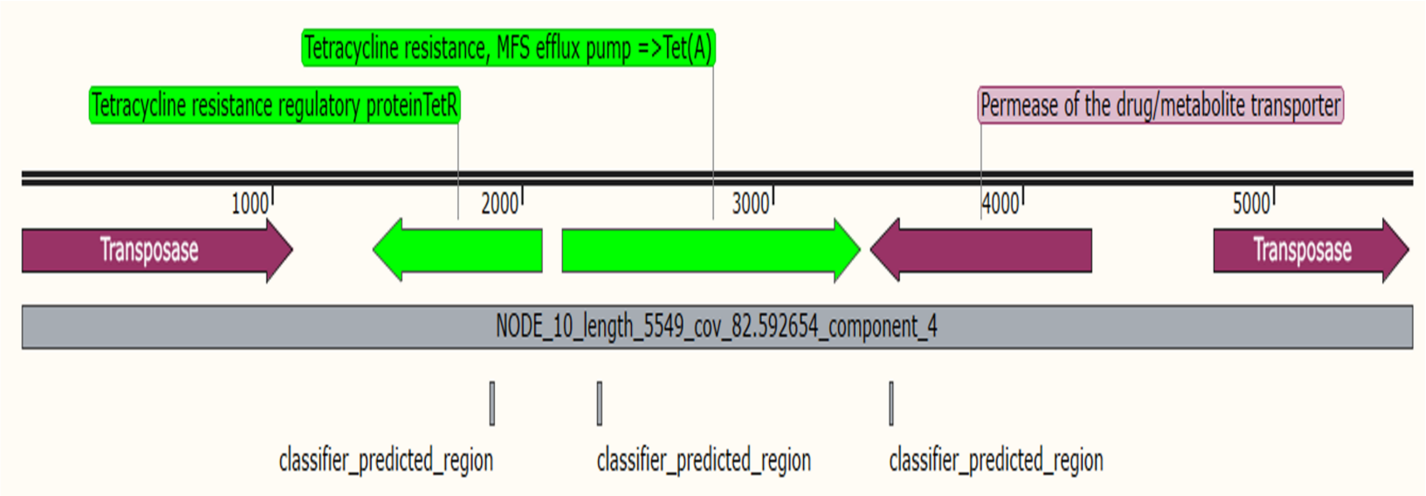

Figure S5. Map of *K. pneumoniae* plasmid *p*MDR; the black horizontal lines indicates the length of the plasmid, the gray line contains information about plasmid length and coverage, the purple arrows indicate transposases and the green arrows represents tet(A) MFS family efflux pump and *TetR* regulatory gene.

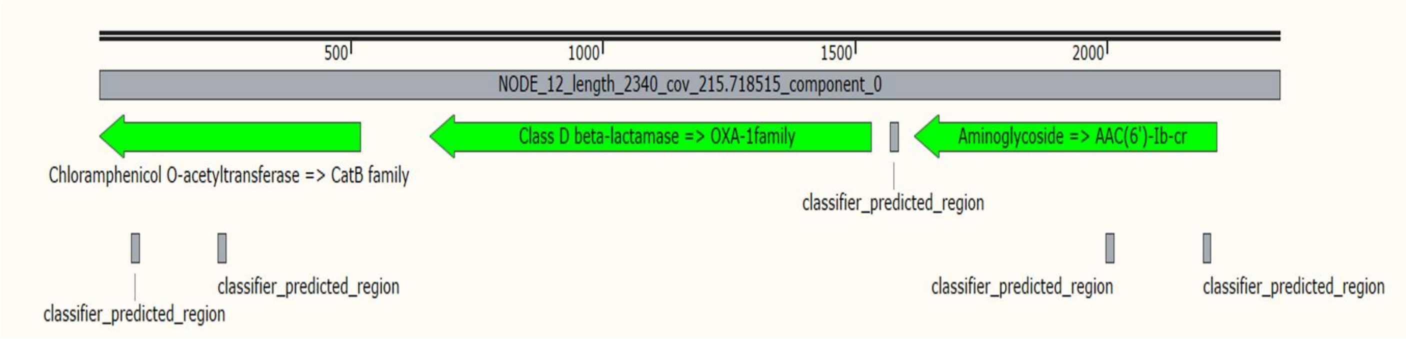

Figure S6. Map of *K. pneumoniae* plasmid *p*3K157; the black horizontal lines indicate the length of the plasmid, the gray line contains information about plasmid length and coverage, and the green arrows represent the ARGs

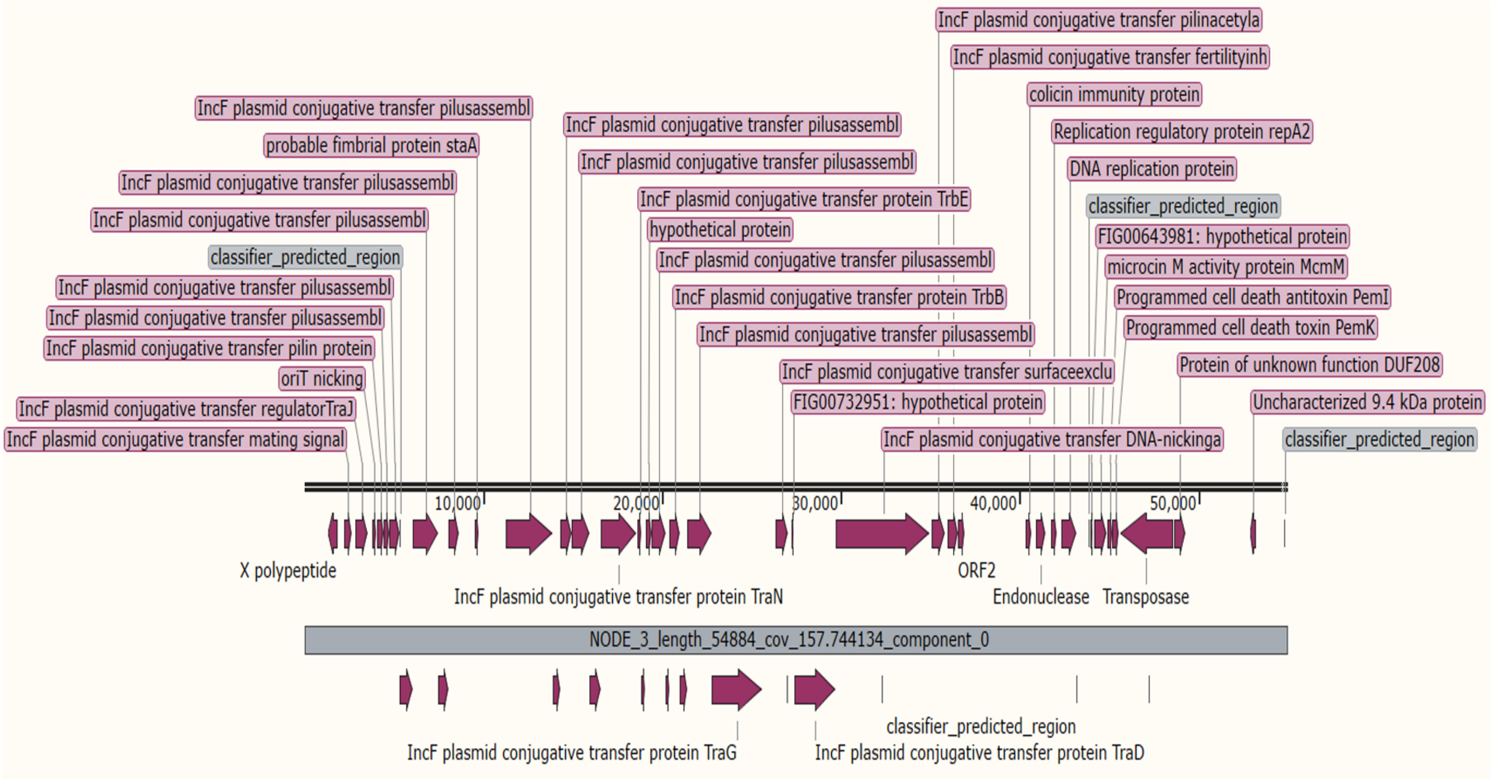

Figure S7. Map of *K. pneumoniae* strain 9PK plasmid *p*1_020143, the horizontal line indicates the length of the plasmid, the middle gray line contains information about plasmid length, and coverage, and the purple arrows contain IncF related genes.
